# Negative decision outcomes are more common among people with lower decision-making competence: an item-level analysis of the Decision Outcome Inventory (DOI)

**DOI:** 10.3389/fpsyg.2015.00363

**Published:** 2015-04-07

**Authors:** Andrew M. Parker, Wändi Bruine de Bruin, Baruch Fischhoff

**Affiliations:** ^1^RAND CorporationPittsburgh, PA, USA; ^2^Centre for Decision Research, Leeds University Business School, University of LeedsLeeds, UK; ^3^Department of Engineering and Public Policy, Carnegie Mellon UniversityPittsburgh, PA, USA; ^4^Department of Social and Decision Sciences, Carnegie Mellon UniversityPittsburgh, PA, USA

**Keywords:** decision-making, competence, outcomes, life events, individual differences

## Abstract

Most behavioral decision research takes place in carefully controlled laboratory settings, and examination of relationships between performance and specific real-world decision outcomes is rare. One prior study shows that people who perform better on hypothetical decision tasks, assessed using the Adult Decision-Making Competence (A-DMC) measure, also tend to experience better real-world decision outcomes, as reported on the Decision Outcomes Inventory (DOI). The DOI score reflects avoidance of outcomes that could result from poor decisions, ranging from serious (e.g., bankruptcy) to minor (e.g., blisters from sunburn). The present analyses go beyond the initial work, which focused on the overall DOI score, by analyzing the relationships between specific decision outcomes and A-DMC performance. Most outcomes are significantly more likely among people with lower A-DMC scores, even after taking into account two variables expected to produce worse real-world decision outcomes: younger age and lower socio-economic status. We discuss the usefulness of DOI as a measure of successful real-world decision-making.

## Introduction

Throughout their lives, people face decisions that affect their finances, health, and overall quality of life. Behavioral decision research aims to understand how people make decisions, with the ultimate goal of improving experienced decision outcomes. However, examination of these real-world decision outcomes is rare. As an experimental science, behavioral decision research uses laboratory settings deliberately isolated from everyday decisions. The hope is that tasks designed to capture fundamental properties of those decisions will predict real-world decision performance and outcomes. In Parker and Fischhoff (2005), we examined whether adolescents who did better on commonly used laboratory decision tasks also did better on presumptive precursors and consequences of good decision-making. We found good construct validity, a pattern that has borne up in a unique longitudinal data collection effort that followed the sample for 11 years (Parker et al., 2014). Other research has also linked performance on decision-making competence tasks to various cognitive abilities (e.g., Stanovich and West, 2000, 2008; Peters et al., 2006; Finucane and Gullion, 2010 Del Missier et al., 2012, 2013), need for cognition and other cognitive styles (e.g., Carnevale et al., 2011; Smith and Levin, 1996), less regret (Parker et al., 2007), and fewer suspensions among students (Stanovich et al., 2003). Moreover, decision-making competence has been improved as a result of targeted decision education among high-school students (Jacobson et al., 2012).

In Bruine de Bruin et al. (2007), we adopted complementary strategies for assessing decision-making competence and outcomes with adults. We first developed a composite measure of Adult Decision-Making Competence (A-DMC), using a set of hypothetical decision tasks drawn from behavioral decision research. We then validated performance on A-DMC against a composite score of decision outcomes as self-reported on the Decision Outcomes Inventory (DOI), which can be accessed through the online Decision-Making Individual Differences Inventory^1^.

The DOI elicits self-reports of outcomes that could result from poorly made decisions. They range from serious (e.g., declared bankruptcy) to minor (e.g., got blisters from sunburn). The DOI was patterned after “life events” scales, which assess individuals' overall life stress through their self-reports of life events found to have adverse consequences (Masten et al., 1994; Brady and Matthews, 2002). We used a 10-year time frame to allow time for severe, and relatively uncommon, events to happen. Most items were preceded by screening questions (have you ever had a credit card?), establishing the possibility of experiencing a bad outcome if a poor decision were made (have you ever had $5000 in credit card debt?). The overall DOI score reflects avoiding more negative decision outcomes, such that higher scores reflect better performance. In Bruine de Bruin et al. (2007), we found that people who did better on hypothetical decision tasks (as seen in high A-DMC scores) also experienced better real-world decision outcomes (as captured by the DOI score).

The approach to DOI scoring in Bruine de Bruin et al. (2007) assumed that, across time, people, and decisions, good decision processes would predict good decision outcomes *on average*. That said, even the soundest decision-making processes cannot guarantee good outcomes. In uncertain situations, some unhappy surprises are inevitable even to good decision makers. Moreover, disadvantaged socio-economic backgrounds may increase exposure to negative life events (Brady and Matthews, 2002). We found a significant relationship between decision-making competence (as measured on A-DMC) and better decision outcomes (as reported on the DOI), even after controlling for socio-economic status (Bruine de Bruin et al., 2007).

Because the DOI was designed to capture a range of decision outcomes varying in severity, the question arises how much each contributed to the relationship between A-DMC and DOI. There are several reasons to suspect that some of the negative outcomes listed on the DOI might not be correlated to decision-making competence (or A-DMC). Specifically, it is possible that some negative outcomes actually reflect the best decision that can be made in a bad situation. Some outcomes are bad in one respect, but good in another. Moreover, some outcomes may not reflect one's own decision making. For example, getting divorced may be the best outcome for a bad marriage, be instigated by one's spouse, and involve a mix of positive experiences (i.e., starting anew) and negative ones (i.e., regret about having chosen a spouse poorly). Answering these concerns bears both on the interpretation of the DOI—is it capturing a unified construct of poor decision outcomes—and on future development of the DOI—item-level analysis could help inform future refinement or reduction in length.

Here, we go beyond our previous analyses and examine the relationships between A-DMC scores and the individual outcomes listed on the DOI. We also control for age and socio-economic status (SES), both potentially correlated with individuals' exposure to the possibility of negative events and ability to keep them from happening. Younger individuals may make more poor decisions because they have had less opportunity to acquire experience and may have greater struggles with impulsivity and social pressures (Reyna and Farley, 2006). Lower SES could force individuals to confront harder decisions, hence creating more opportunities for negative outcomes, and to have fewer resources for mitigating their effects (Brady and Matthews, 2002). In Parker and Fischhoff (2005), we found evidence suggesting that young people growing up in lower SES environments may have less opportunity to observe sound decision making and receive less consistent positive reinforcement for their own attempts to master those skills.

## Methods

We re-analyzed data collected from 360 participants, recruited from the Pittsburgh community through social-service and other community organizations (Bruine de Bruin et al., 2007). This strategy created a sample that varied widely in socio-economic status (which we assessed with the proxy measure of the social-service organizations through which we recruited participants). Respondents ranged in age from 18 to 88, 74% were women, 66% self-identified as White, 28% as African American, and 6% as other. Forty percent reported having at least a bachelor's degree.

In group-administered sessions, participants completed both the DOI and the A-DMC battery. The DOI asked them to self-report on the 41 negative decision outcomes in Table 1. Thirty-five of these outcomes were preceded by filter questions asking if participants had been in a position to experience the outcome. For example, the question on whether the participant had missed a flight (the outcome) was preceded by a question asking whether they had taken a plane trip (the filter). Overall DOI scores were computed as the percentage of negative outcomes experienced, among those that could have happened^2^.

**Table 1:** **Outcomes captured by the Decision-Outcomes Inventory for low and high A-DMC subsamples (among those for whom each was possible)**.

**DOI outcome**	**Prevalence of DOI outcome**		
	**% in Low A-DMC**	**% in High A-DMC**	**Difference**
Had more than $5000 in credit card debt	30.1	43.6	−13.5^*^
Gotten lost or gone the wrong way for more than 10 min while driving	60.5	70.6	−10.1^*^
Returned a movie you rented without having watched it at all	72.1	77.2	−5.1
Had a check bounce	37.0	41.0	−4.0
Threw out food or groceries you had bought, because they went bad	88.1	91.3	−3.2
Lost more than $1000 on a stock-market investment	46.4	48.9	−2.5
Got blisters from sun burn	25.0	27.3	−2.3
Forgotten a birthday of someone close to you and did not realize until the next day or later.	56.8	57.9	−1.1
Taken the wrong train or bus	15.3	13.1	2.2
Loaned more than $50 to someone and never got it back	70.1	67.7	2.4
Bought new clothes or shoes you never wore	59.7	56.5	3.2
Locked your keys in the car	56.1	52.5	3.6
Been diagnosed with type 2 diabetes	9.5	4.7	4.8^+^
Consumed so much alcohol you vomited	41.2	35.8	5.4
Received a DUI for drunk driving	8.2	2.7	5.5^+^
Had your driver's license taken away from you by the police	11.6	5.6	6.0^+^
Been accused of causing a car accident while driving	21.0	14.7	6.3
Declared bankruptcy	12.2	5.9	6.3^*^
Broke a bone because you fell, slipped, or misstepped	20.3	13.5	6.8^+^
Gotten more than 5 speeding tickets	8.1	1.2	6.9^**^
Locked yourself out of your home	48.3	41.0	7.3
Missed a flight	14.7	7.3	7.4^+^
Been in a public fight or screaming argument	24.0	16.5	7.5^+^
Gotten more than 5 parking tickets	13.0	4.9	8.1^*^
Ruined your clothes because you didn't follow the laundry instructions on the label	55.1	46.0	9.1^+^
Been diagnosed with an STD (Sexually Transmitted Disease)	13.1	3.4	9.7^**^
Foreclosed a mortgage or loan	12.9	2.9	10.0^**^
Been in a jail cell overnight for any reason	13.1	2.9	10.2^***^
Paid a rent or mortgage payment at least 2 weeks too late	31.6	19.4	12.2^*^
Been kicked out of a bar, restaurant, or hotel by someone who works there	15.7	3.0	12.7^***^
Had a condom break, tear, or slip off	39.1	25.7	13.4^+^
Had an unplanned pregnancy (or got someone pregnant, unplanned)	24.5	9.9	14.6^**^
Had to spend at least $500 to fix a car you had owned for less than half a year	41.6	26.8	14.8^*^
Been suspended from school for at least 1 day for any reason	24.4	7.9	16.5^**^
Been divorced	26.2	9.3	16.9^**^
Had your ID replaced because you lost it	32.6	15.6	17.0^***^
Been kicked out of an apartment or rental property before the lease ran out	23.6	3.4	20.2^***^
Quit a job after a week	23.2	2.6	20.6^***^
Had the key to your home replaced because you lost it	35.0	13.2	21.8^***^
Had your electricity, cable, gas or water shut off because you didn't pay on time	27.9	4.6	23.3^***^
Cheated on your romantic partner of 1 year by having sex with someone else	34.9	7.2	27.7^***^
Mean percentage of outcomes	32.3%	24.8%	7.5%^***^

Difference in prevalence tested using chi-square test of independence for individual outcomes and using two-sample t-test for mean percentage;

+ p < 0.10;

* p < 0.05;

** p < 0.01;

*** *p < 0.001*.

*The final six items had no conditioning event*.

A-DMC has six hypothetical decision tasks: Resistance to Framing, Recognizing Social Norms, Under/overconfidence, Applying Decision Rules, Consistency in Risk Perception, and Resistance to Sunk Costs (the full instrument can be found at the online Decision-Making Individual Differences Inventory^3^). Responses are scored in terms of either consistency with one another or correspondence to an external criterion. Here we use a summary A-DMC score, equal to an unweighted mean of the standardized task scores, standardized to have zero mean and standard deviation of one. We use a median split in Table 1 as an easy way to present the differences in performance among individuals with higher and lower scores.

This research was approved by the Carnegie Mellon University Institutional Review Board, and all data collection conformed to U.S. human subjects regulatory standards. Informed consent was obtained from all subjects.

### Analysis plan

We first summarize the rate of experiencing each DOI outcome for those with relatively low and high A-DMC (Table 1). We then (in Table 2) use bivariate and multivariate logistic regression to predict each DOI outcome and the overall DOI score with A-DMC (using the full range), age, and SES^4^.

**Table 2:** **Odds of outcomes on the Decision-Outcomes Inventory as a function of A-DMC, age, and SES**.

**DOI outcome**	**Bivariate odds**	**Multivariate odds (with control variables)**		
	**A-DMC**	**A-DMC**	**Age**	**Low SES**
Had more than $5000 in credit card debt	1.43^*^	1.49^*^	0.70^***^	1.16
Gotten lost or gone the wrong way for more than 10 min while driving	1.23	1.16	0.78^**^	0.80
Returned a movie you rented without having watched it at all	1.26	1.12	0.56^***^	0.50^*^
Had a check bounce	1.02	1.28	0.64^***^	2.95^***^
Threw out food or groceries you had bought, because they went bad	1.38^+^	1.46	0.75^**^	1.18
Lost more than $1000 on a stock-market investment	1.12	1.04	1.05	0.69
Got blisters from sun burn	1.24	1.04	0.86^+^	0.49^*^
Forgotten a birthday of someone close to you and did not realize until the next day or later	1.03	1.00	0.84^*^	0.83
Taken the wrong train or bus	0.94	1.05	0.70^*^	1.31
Loaned more than $50 to someone and never got it back	0.89	0.95	0.87	1.25
Bought new clothes or shoes you never wore	0.93	1.06	0.88^+^	1.66^+^
Locked your keys in the car	1.03	0.91	1.12	0.60^+^
Been diagnosed with type 2 diabetes	0.61^*^	0.75	1.27^+^	3.12^+^
Consumed so much alcohol you vomited	0.90	1.02	0.53^***^	0.97
Received a DUI for drunk driving	0.45^**^	0.57	0.87	2.03
Had your driver's license taken away from you by the police	0.48^**^	0.54^*^	0.67^*^	1.42
Been accused of causing a car accident while driving	0.90	0.85	0.87	0.74
Declared bankruptcy	0.55^**^	0.92	0.77^*^	8.43^***^
Broke a bone because you fell, slipped, or misstepped	0.87	0.73^+^	0.91	0.46^*^
Gotten more than 5 speeding tickets	0.26^***^	0.31^**^	0.93	2.11
Locked yourself out of your home	0.84	0.97	0.84^*^	1.75^+^
Missed a flight	0.68	0.75	0.76^+^	1.45
Been in a public fight or screaming argument	0.73^*^	0.86	0.56^***^	0.54
Gotten more than 5 parking tickets	0.54^*^	0.77	0.74^*^	3.75^*^
Ruined your clothes because you didn't follow the laundry instructions on the label	0.89	0.84	0.78^***^	0.78
Been diagnosed with an STD (Sexually Transmitted Disease)	0.45^**^	0.80	0.76	12.62^**^
Foreclosed a mortgage or loan	0.28^***^	0.37^**^	0.96	6.87^**^
Been in a jail cell overnight for any reason	0.43^***^	0.66^+^	0.76^*^	5.07^**^
Paid a rent or mortgage payment at least 2 weeks too late	0.70^*^	0.99	0.76^*^	3.80^***^
Been kicked out of a bar, restaurant, or hotel by someone who works there	0.42^***^	0.52^*^	0.53^***^	1.69
Had a condom break, tear, or slip off	0.65^*^	0.69^+^	0.88	1.18
Had an unplanned pregnancy (or got someone pregnant, unplanned)	0.52^***^	0.74	0.73^*^	3.69^**^
Had to spend at least $500 to fix a car you had owned for less than half a year	0.62^**^	0.61^*^	0.75^**^	0.96
Been suspended from school for at least 1 day for any reason	0.40^***^	0.52^**^	0.69^*^	2.63^+^
Been divorced	0.46^***^	0.61^*^	0.80	4.13^**^
Had your ID replaced because you lost it	0.58^***^	0.78	0.69^***^	3.05^***^
Been kicked out of an apartment or rental property before the lease ran out	0.25^***^	0.28^***^	0.94	3.99
Quit a job after a week	0.31^***^	0.42^**^	0.86	3.12^*^
Had the key to your home replaced because you lost it	0.50^***^	0.66^*^	0.71^***^	3.01^**^
Had your electricity, cable, gas or water shut off because you didn't pay on time	0.38^***^	0.55^*^	0.71^**^	5.72^***^
Cheated on your romantic partner of 1 year by having sex with someone else	0.42^***^	0.64^*^	0.84	5.61^***^
Mean percentage of outcomes	−0.26^***^	−0.15^*^	−0.36^***^	0.19^**^

Effects presented as odds ratios presented from multivariate logistic regressions (for individual outcomes) and standardized linear regression coefficients (for mean percentage);

+ p < 0.10;

* p < 0.05;

** p < 0.01;

*** *p < 0.001*.

*The final six items had no conditioning event*.

This paper is accompanied by supplementary material that provides additional detail on the relationships between individual DOI outcomes and individual A-DMC subscales. The pattern of results broadly support the relationships reported below, as well as correlations of overall DOI with DMC subscales (Bruine de Bruin et al., 2007).

## Results

### How is A-DMC related to individual DOI outcomes?

Table 1 shows the frequency of each negative DOI outcome, for participants with lower and higher DMC scores (among those for whom that outcome was possible), ordered by the difference in percentages. The first eight items are ones for which low A-DMC appears protective, in the sense of being reported less often by low A-DMC participants. The two statistically significant differences (*p* < 0.05) show that low-A-DMC participants are less likely to have more than $5000 credit card debt and to have gotten lost while driving. The other 33 outcomes are less likely for participants with high A-DMC, significantly so in most cases. On average, the outcomes are 7.5% less likely for high A-DMC participants. Thus, if A-DMC is accepted as a measure of decision-making ability, then most DOI outcomes are indicators of poor decision making. Nonetheless, the exceptions are puzzling and perhaps partially explained by the analyses that follow.

### Do DOI outcomes reflect factors other than decision-making competence?

Table 2 considers the effects of two non-decision factors on DOI outcomes: age and SES. The results are odds ratios from logistic regression analyses, using the full range of the A-DMC scores (rather than a binary variable), normalized so that each unit increment represents one standard deviation. The bivariate odds express the difference in the prevalence of the DOI outcome in odds ratios terms, with ratios less than one meaning that the (bad) outcome is more likely for participants with lower A-DMC scores. As would be expected from Table 1, the first eight items have odds ratios greater than one^5^. The next column shows the odds ratio for the A-DMC scores, after controlling for age and SES. The overall pattern is largely unchanged. Three quarters of the odds ratios are less than one, and often significantly so. Nonetheless, most are closer to one than in the previous column, indicating that these controls are related to the decision outcomes.

The final two columns show odds ratios for age and SES, controlling for each other and A-DMC. Age is scaled continuously, with each unit equal to 10 years to show differences better^6^. The odds ratios for age are almost all less than one, indicating that the DOI outcomes are more likely for younger respondents (as mentioned, controlling for A-DMC and SES). For example, the younger respondents are 14% (1/0.88 – 1) more likely to report having bought and never worn clothing or shoes in the preceding 10 years (the reporting period for these items) and almost twice as likely to have been kicked out of a bar, restaurant, or hotel (the smallest ratio). In the final column (Low SES), about three-quarters of the odds ratios are greater than one, indicating that the outcome is more likely for lower-SES individuals. For example, they are 66% more likely not to have worn shoes or clothing that they bought (row 1) and almost 13 times more likely to report having been diagnosed with an STD (the highest ratio) (controlling for A-DMC and SES).

The final row of Table 2 presents standardized linear regression coefficients for predicting the overall percentage of negative outcomes that participants reported experiencing (among those possible for each individual) with A-DMC, age, and SES. The results show the strongest effect for age, with both A-DMC and SES showing significant marginal effects. However, controlling for SES has a much stronger impact on the A-DMC coefficient than does controlling for age, as A-DMC correlates strongly with SES (*r* = −0.52, *p* < 0.001) but not with age (*r* = 0.04, ns).

## Discussion

Here, we set out to address concerns about whether or not individual items on the Decision Outcome Inventory (DOI) are indicative of decision-making competence. We found that most of the DOI outcomes are significantly more likely among participants with lower A-DMC scores, suggesting that these outcomes reflect, in part, poor decision-making competence. Many of these outcomes are also related to two variables that would be expected to produce worse outcomes: being young and being poor. Indeed, most DOI outcomes are more common among younger and lower SES participants. Young people have had less opportunity to learn how to make decisions. Lower SES individuals may face more perilous circumstances that expose them to higher chances of negative outcomes (e.g., taking risks to avoid crime or save money). Both may have fewer resources for limiting damage (e.g., legal representation). Parker and Fischhoff (2005) found that lower SES young adults had lower decision-making competence for reasons potentially related to their opportunities to acquire those skills (e.g., stable home environment). Thus, SES may affect both the decisions that individuals face and their ability to cope with them.

If age and SES are viewed as factors outside of individuals' control, then the correlation between DOI outcomes and A-DMC after controlling for them might indicate the degree to which those outcomes are under individuals' control. In that light, being evicted, quitting a job after a week, being suspended from school, getting five or more speeding tickets, and having a mortgage or loan foreclosed appear to be the bad outcomes that good decision making can do the most to avoid. These may also be the individual outcomes for which DOI has the clearest interpretation as reflecting poor decision-making competence.

Age has the strongest independent relationship to DOI, even after taking into account potential differences in A-DMC and SES. We found age differences for several outcomes: having a large credit card debt, having a check bounce, drinking enough alcohol to vomit, ruining clothes by not following laundry instructions, and being in a public fight or screaming argument Each of these outcomes may reflect the higher risks faced by younger people. Low SES has the strongest independent relationship to DOI (controlling for A-DMC and age) for outcomes involving economic problems potentially outside individuals' control, where decision-making competence cannot overcome circumstantial constraints: having a check bounce, declaring bankruptcy, and being behind in rent or mortgage payments. Those are followed by having a mortgage or loan foreclosed, being in jail overnight, being diagnosed with an STD, and having an unplanned pregnancy.

### Suggestions for future research

The DOI was developed as an outcome assessment that (a) is convenient for administration in paper-and-pencil or web-based formats and (b) targets likely results of poor decision-making processes. The present analyses support its validity in two ways. One is that most of the individual outcomes are more common among individuals with lower scores on A-DMC. The second is that poor outcomes are more common among younger people and those from lower SES backgrounds, in predictable ways.

Although most outcomes show this pattern, some do not. One possible way to improve the measure is by reducing it to those items that performed best. A second is to refine the items that performed most poorly. For example, the 10-year reporting period may have taken younger respondents into time periods for which the outcome was irrelevant (e.g., accumulating $5000 in credit card debt while in high school). A related response is to create items targeting specific populations (e.g., fraud victimization for the elderly). Similarly, future work could examine domain-specific item sets (e.g., financial or health), similar to methods used in developing the Domain-Specific Risk-Taking (DOSPERT) scale (Blais and Weber, 2006). We would like to see the present analyses repeated with new samples, new items, and additional covariates having plausible relationships to decision outcomes. We would also like to see a complementary scale with good outcomes. Finally, DOI relies on the accuracy (and candor) of people's self-reports of negative outcomes^7^. We were surprised by how many poor outcomes participants reported. We speculate that having so many things that could go wrong made it easier to admit some as personal failings. Nonetheless, independent validation of some outcomes would be valuable, perhaps using existing panel studies that collect diverse data on health, finances, and other domains. Despite these limitations, the DOI, in whole or in part, may provide an efficient way to assess participants' success in decision making, given the constraints of their age and SES.

## Author contributions

All three authors contributed to the design, data acquisition, analysis, interpretation, and writing for the research described here.

### Conflict of interest statement

The authors declare that the research was conducted in the absence of any commercial or financial relationships that could be construed as a potential conflict of interest.

## Footnotes

^1^http://www.sjdm.org/dmidi/Decision_Outcome_Inventory.html

^2^Here, we focus on the unweighted percentage, which is highly correlated with the weighted percentage (*r* = 0.92, *p* < 0.001) also reported by Bruine de Bruin et al. (2007), with which outcomes are weighted by the reciprocal of their frequency—assuming that rarer outcomes are worse. Hence, here overall DOI can be thought of as a simple summary of the 41 individual outcomes, rather than one emphasizing uncommon (and more severe) outcomes.

^3^http://www.sjdm.org/dmidi/Adult_-_Decision_Making_Competence.html

^4^We also considered gender, but chose to leave it out of the reported analyses, since we did not have strong a priori expectations for a direction of effect. That said, the results for gender were quite modest, especially considering the number of tests. In total, six outcomes (suspended from school, lost driver's license, kicked out of bar, drank so much you vomited, DUI, and public fight) were more likely among men (at *p* < 0.05) and only one outcome (bought new clothes or shoes you never wore) was more likely among women.

^5^The tests result in slightly different significance levels than in Table 1, largely due to the use of the full range of the A-DMC variable, rather than a binarized version.

^6^The marginal effect of a single year in age is quite small, hence we divided age by 10 to provide odds ratios that are easier to interpret.

^7^In contrast, A-DMC is a performance metric, and is therefore less susceptible to self-report biases. Hence, the fact that the two correlate, without shared method variance, adds support to the validity of the DOI responses.
