# Supplementary material for: Negative decision outcomes are more common among people with lower decision-making competence: an item-level analysis of the Decision Outcome Inventory (DOI)
Source: Front Psychol. 2015 Apr 7;6:363. doi: 10.3389/fpsyg.2015.00363 (PMC4387469; doi:10.3389/fpsyg.2015.00363)
Supplement: Supplementary file 1 [file DataSheet1.PDF]

Negative decision outcomes are more common among people with lower decision-making competence:

An item-level analysis of the Decision Outcome Inventory (DOI)

Supplementary Material

Table S1 provides further detail on Table 1 in the main paper, by presenting pairwise associations between A-DMC subscales and individual DOI outcomes (Bruine de Bruin et al., 2007), as well as mean percentage of DOI outcomes. Items are ordered as in Table 1. Reported statistics are odds ratios derived from individual logistic regressions predicting individual DOI outcomes with individual A-DMC subscales. In the final row (mean percentage of DOI outcomes), statistics are standardized beta coefficients from linear regressions.

These data are provided as a resource to readers. Caution should be taken in interpreting individual coefficients, given the large number of tests conducted. We envision that the results as useful for identifying general trends (e.g., many of the relationships to overall A-DMC, as seen in Table 1 of the main paper, bear out across the individual A-DMC subscales) and for future scale refinement or reduction (e.g., some outcomes show more consistent negative relationships with A-DMC).

Table S1. Odds ratios from logistic regressions predicting individual DOI items (and total score) from A-DMC subscales (and total score).

DOI Item #	DOI Outcome	Resistance to Framing	Resistance to Sunk Cost	Applying Decision Rules	Under/ over-confidence	Consistency in Risk Perception	Recognizing Social Norms
19	Had more than $5000 in credit card debt	1.48**	1.12	1.20	1.07	1.34*	1.18
8.4	Gotten lost or gone the wrong way for more than 10 minutes while driving	1.29	.82	1.25	.94	1.14	1.45*
1	Returned a movie you rented without having watched it at all	1.36*	.75*	1.54**	1.28	1.17	1.24
18	Had a check bounce	1.29	.85	1.04	.97	1.17	.88
3	Threw out food or groceries you had bought, because they went bad	1.41*	.82	1.38	1.10	1.33	1.10
20	Lost more than $1000 on a stock-market investment	1.23	.92	1.36	1.03	1.06	.86
28	Got blisters from sun burn	1.24	.77	1.31	1.24	1.03	.96
32	Forgotten a birthday of someone close to you and did not realize until the next day or later.	1.00	1.09	1.09	.98	.84	1.03
11	Taken the wrong train or bus	1.62*	.77	.98	1.02	.90	.58**
22	Loaned more than $50 to someone and never got it back	1.24	1.00	1.04	.86	.96	.57**
2	Bought new clothes or shoes you never wore	1.24	.96	.90	.92	.90	.89
8.5	Locked your keys in the car	.97	.93	1.04	.92	.99	1.21
33	Been diagnosed with type 2 diabetes	.64*	.85	.51*	.86	.67	.71
27.1	Consumed so much alcohol you vomited	1.05	.63**	1.13	.99	1.01	.77
27.2	Received a DUI for drunk driving	.67	.68	.38**	.61*	.65	.50*
7	Had your driver's license taken away from you by the police	1.05	.61*	.62*	.84	.68	.34***
8.1	Been accused of causing a car accident while driving	1.00	.77	.94	1.12	.95	.88
31	Declared bankruptcy	.77	.65*	.57*	.83	.53***	.69*
34	Broke a bone because you fell, slipped, or misstepped	.95	.97	.83	.88	.84	.82
8.3	Gotten more than 5 speeding tickets	.73	.40**	.29**	.85	.54*	.32***
14.2	Locked yourself out of your home	1.01	.85	.90	1.02	.83	.85
10	Missed a flight	1.12	.95	.65	.80	.55**	.63
30	Been in a public fight or screaming argument	.95	.63**	.85	.80	.94	.65**
8.2	Gotten more than 5 parking tickets	.86	.61*	.54**	.61*	1.16	.56*
4	Ruined your clothes because you didn't follow the laundry instructions on the label	1.09	.71**	.88	.87	.94	.80
25.1	Been diagnosed with an STD (Sexually Transmitted Disease)	.91	.64	.48**	.61*	.62*	.44***
16	Foreclosed a mortgage or loan	.67	.61	.25***	.33***	.52*	.35**
29	Been in a jail cell overnight for any reason	.76	.70	.34***	.64*	.55**	.46***
17	Paid a rent or mortgage payment at least 2 weeks too late	.82	.70*	.80	.76	.92	.79
21	Been kicked out of a bar, restaurant, or hotel by someone who works there	.69	.55**	.37***	.72	.75	.53**
26	Had a condom break, tear, or slip off	.58**	.95	.66*	.76	.88	.63*
25.2	Had an unplanned pregnancy (or got someone pregnant, unplanned)	.77	.72	.60**	.69*	.60**	.53***
9	Had to spend at least $500 to fix a car you had owned for less than half a year	.84	1.02	.73*	.54**	.95	.69*
5	Been suspended from school for at least one day for any reason	.72	.55**	.43***	.49***	.56**	.51**
24	Been divorced	.84	.89	.43***	.50**	.57**	.81
12	Had your ID replaced because you lost it	1.00	.68**	.67**	.96	.74*	.67*
13	Been kicked out of an apartment or rental property before the lease ran out	.70	.48**	.24***	.54**	.47**	.55*
6	Quit a job after a week	.72	.46***	.49**	.72	.59**	.43***
14.1	Had the key to your home replaced because you lost it	.91	.79	.56***	.81	.62***	.52***
15	Had your electricity, cable, gas or water shut off because you didn't pay on time	.78	.67*	.36***	.59**	.59**	.70*
23	Cheated on your romantic partner of 1 year by having sex with someone else	.70	.73	.40***	.44***	.61*	.48***
	Mean percentage of outcomes	*-.01*	*-.21****	*-.21****	*-.15***	*-.16***	*-.24****

Note: Statistics are odds ratios from logistic regressions predicting DOI outcomes from A-DMC total score and subscores. For mean percentage of outcomes row, statistics are standardized beta coefficients from linear regressions. * p-value < .05; ** p < .01; *** p < .001

References

Bruine de Bruin, W., Parker, A. M., & Fischhoff, B. (2007). Individual differences in Adult Decision-Making Competence. *Journal of Personality and Social Psychology*, *92*, 938-956.
